# Novel Peptides Targeting the β-Clamp Rapidly Kill Planktonic and Biofilm *Staphylococcus epidermidis* Both *in vitro* and *in vivo*

**DOI:** 10.3389/fmicb.2021.631557

**Published:** 2021-03-17

**Authors:** Synnøve Brandt Raeder, Erik Thorvaldsen Sandbakken, Anala Nepal, Kirsti Løseth, Kåre Bergh, Eivind Witsø, Marit Otterlei

**Affiliations:** ^1^Department of Clinical and Molecular Medicine, Faculty of Medicine and Health Sciences, Norwegian University of Science and Technology (NTNU), Trondheim, Norway; ^2^Department of Orthopaedic Surgery, St. Olav University Hospital, Trondheim, Norway; ^3^Department of Medical Microbiology, St. Olav University Hospital, Trondheim, Norway

**Keywords:** prosthetic joint infections, antimicrobial peptides, APIM-peptide, antimicrobial resistance, antibiotic

## Abstract

Antimicrobial resistance is an increasing threat to global health and challenges the way we treat infections. Peptides containing the PCNA interacting motif APIM (APIM-peptides) were recently shown to bind to the bacterial PCNA homolog, the beta (β)-clamp, and to have both antibacterial and anti-mutagenic activities. In this study we explore the antibacterial effects of these peptides on *Staphylococcus epidermidis*, a bacterial species commonly found in prosthetic joint infections (PJI). Drug-resistant bacterial isolates from PJIs often lead to difficult-to-treat chronic infections. We show that APIM-peptides have a rapid bactericidal effect which when used at sublethal levels also increase the efficacy of gentamicin. In addition, APIM-peptides reduce development and eliminate already existing *S. epidermidis* biofilm. To study the potential use of APIM-peptides to prevent PJI, we used an *in vivo* bone graft model in rats where APIM-peptide, gentamicin, or a combination of the two was added to cement. The bone grafts containing cement with the combination was more effective than cement containing only gentamicin, which is the current standard of care. In summary, these results suggest that APIM-peptides can be a promising new drug candidate for anti-infective implant materials to use in the fight against resistant bacteria and chronic PJI.

## Introduction

Infections are considered a serious clinical problem for implant surgery and new solutions for anti-infective implant materials are needed (Arciola et al., 2018). *Staphylococcus aureus* and *S. epidermidis* are known to be nosocomial bacterial species and the most common species isolated from orthopedic implant infections (Malhas et al., 2015). Both bacteria are part of our microbiota and are normally not associated with disease. *S. aureus* is a frequently isolated pathogen in humans while infections by *S. epidermidis* are less common. However, introduction of *S. epidermidis* to surgical implants or to immunocompromised patients can cause chronic, difficult-to-treat infections in contrast to the more acute *S. aureus* infections (Otto, 2014).

Orthopedic implant infections with antibiotic-resistant bacteria usually have a worse outcome than antibiotic sensitive infections, and often leads to implant removal and long-term antibiotic therapies (Li and Webster, 2018). Difficult-to-treat PJIs are therefore devastating events for patients (Oliveira et al., 2018). Vancomycin has been the last option antibiotic for treating infections with drug-resistant Gram-positive bacteria like methicillin-resistant *S. aureus* and *S. epidermidis* (MRSA and MRSE) (Al-Tatari et al., 2006). However, vancomycin resistance has also been detected in *S. epidermidis* clinical isolates (Ahanotu et al., 2001; Pahadi et al., 2014). Long term exposure to antibiotics at sub-lethal concentrations can create selective pressure on a bacterial population. Mutagenesis triggered by antibiotic-induced stress can be a driver for the development of resistance (Kreuzer, 2013). The prophylactic use of antibiotic-loaded bone cement can therefore contribute to more resistance development. Incidentally, the prevalence of gentamicin-resistant infections has increased after bone cement loaded with gentamicin was put to use (Hanssen, 2004).

The ability of *S. epidermidis* to form biofilm is important for its pathogenicity and biofilm formation often leads to a poorer outcome for the patients (Arciola et al., 2005, 2018). Bacteria in biofilms can be less susceptible to antibiotic agents as they are covered in a protective layer of extracellular polymeric matrix. The biofilm can reduce the diffusion of a drug across the matrix, inhibiting the drug to penetrate the “full-depth” of the biofilm. In addition, biofilms make it possible for the bacteria to evade the immune system of the host. Some bacteria in a biofilm are often in a reduced metabolic state, making them less susceptible to antibiotic agents (Costerton et al., 1999). It has been suggested that aminoglycosides can induce biofilm formation (Hoffman et al., 2005). Biofilm-producing *S. epidermidis* from orthopedic implant infections show a significantly higher resistance toward several antibiotics compared to non-biofilm forming strains (Arciola et al., 2005; Sahal and Bilkay, 2014).

Cell penetrating peptides containing APIM, a binding motif for the mammalian DNA sliding clamp PCNA, increase the efficacy of multiple anti-cancer drugs (Muller et al., 2013; Gederaas et al., 2014; Sogaard et al.,2018a,b), and reduce the mutagenesis in mammalian cells (Raeder et al., 2018). These APIM-peptides were recently shown to have antibacterial activities due to binding to the conserved bacterial DNA sliding clamp, the β-clamp, thereby inhibiting DNA replication and mutagenesis. Antibacterial effects were found for several bacterial species, including *Escherichia coli*, *S. epidermidis*, and *S. aureus* (Nedal et al., 2020). Here we explored the possibility of prophylactically using APIM-peptide, in combination with the commonly used antibiotic gentamicin, in bone cement to limit the frequency of PJIs. We found that APIM-peptide had a bactericidal effect on both a gentamicin susceptible and a gentamicin resistant strain of *S. epidermidis* and that APIM-peptide and gentamicin had additive effect. The combination of gentamicin and APIM-peptide in bone cement reduced the growth of gentamicin resistant *S. epidermidis* in bone grafts compared to gentamicin only. In addition, APIM-peptide inhibits biofilm formation at sub-MIC concentrations, and eradicates existing biofilm at ∼8x MIC. Altogether, these results show that the use of APIM-peptides, both alone and in combination with other antibacterial drugs, could be promising for inhibiting PJIs.

## Materials and Methods

### Bacterial Strains and Culture

Two clinical *S. epidermidis* isolates were used (Department of Microbiology, St. Olavs University Hospital, Trondheim, Norway): one gentamicin susceptible *S. epidermidis* and one Methicillin-resistant *S. epidermidis* (MRSE) strain. The MRSE strain is resistant to gentamicin, erythromycin, cloxacillin/dicloxacillin/penicillin. *S. epidermidis* strain ATCC35984 and ATCC12228 were used as a strong and poor biofilm forming strain, respectively. *S. epidermidis* strains were cultured in tryptic soy broth (TSB, 1% glucose) or Mueller-Hinton Broth (MHB) for MIC and Time-killing assay. Blood agar plates were utilized for plating and incubated at 37°C with 5% CO_2_.

### APIM-Peptides

APIM-peptides (Innovagen, Sweden) used here were TFA-salt (>90% pure). The APIM-peptide consists of the APIM-motif (in bold) connected via a linker to an arginine tail (cell penetrating part), containing 10 or 11 arginine; Ac-MD-**RWLVK**-WKKKRKI-R11/R10 the C-termini on all peptides were amidated (Nedal et al., 2020). Lyophilized peptide from the manufacturer was dissolved in water.

### Minimum Inhibitory Concentration Assay

Minimal inhibitory concentration (MIC) was determined following the protocol recommended by National Committee of Laboratory Safety and Standards, NCCLS (NCCLS., 2012) for microtiter broth dilution assay, conducted with modifications suggested by R.E.W. Hancock Lab for cationic peptides^1^. Briefly, bacterial suspension in MHB was adjusted to 0.5 McFarland and diluted to 5 × 10^5^ colony forming units (CFU)/mL. 0.1 mL of the suspension (5 × 10^4^ CFU) was seeded in each well in polypropylene microtiter plates (Greiner). APIM-peptide and gentamicin were added as 10% of the total volume, diluted to specific concentrations (not only twofold serial dilution). After 24 h of incubation (37°C), the plates were inspected for visible growth.

### Time-Killing Assay

Time-kill curves were conducted after NCCLS standards (NCCLS., 1999) by inoculating the MRSE strain in 10 mL MHB, 5 × 10^5^CFU/mL. The cultures were treated at 0 h with 0.5x, 1x, 2x, and 5x MIC concentrations of APIM-peptide (MIC = 5 μM), sub-MIC dose of gentamicin (39 μM, MIC = 309 μM) or 5x MIC vancomycin (10 μM, MIC = 2 μM). 0.5x MIC of APIM-peptide was also combined with gentamicin. The cultures were grown in a shaking incubator (250 rpm, 37°C). Samples were harvested at the time points from 5 min up to 48 h, and plated (duplicates) on blood agar plates in 10-fold dilutions in saline solution (0.9% NaCl). CFUs were counted after 24 h of incubation. The detection limit is calculated to 50 CFU/mL (1.72 log_1__0_CFU/mL); however, zero CFU were included to calculate average CFU counts when no colonies was detected in non-diluted culture. The culture density after media exchange was determined for the cultures added x5 MIC with APIM-peptide/vancomycin at the 48 h timepoint to quantify CFU/mL after the removal of the antibacterial agent. In this case, 5 mL of the cultures were centrifuged (2 min, 14,000 rpm) and the pellet re-suspended in fresh MHB (same volume) before plating.

### *In vitro* Biofilm Formation Assay

Biofilm was quantified following a modified version of the Christensen’s microtiter plate test (Stepanovic et al., 2000). Briefly, *S. epidermidis* strain ATCC35984 were seeded in suspension (200 μL/well) adjusted to 0.5 McFarland turbidity standard in TSB in microtiter plates. APIM-peptide was added directly to the culture, 10% of the total volume, to minimum 3 wells per sample. After 24 h incubation 37°C, the wells were washed and vortexed (3 × 1 min, 600 rpm) with saline solution before fixation with 99% methanol for 15 min. The wells were emptied and dried followed by staining (5 min) with crystal violet (0.5–2%). Wells were washed with water and dried before the stain was re-solubilized with 160 μL glacial acetic acid (33%). Finally, absorbance was measured at 570 nm. ATCC12228 and MRSE (clinical isolate) was also tested with this assay to compare biofilm forming ability (Supplementary Figure S2).

### *In vitro* Biofilm Killing Assay

Biofilm killing assay was performed as previously described (Mandell et al., 2017) with some minor modifications. Briefly, stainless steel wire (C-WIRE, 0.89 mm, ConMed) was cut to 6 mm rods and autoclaved. The rods were placed in 200 μL bacterial suspension with *S. epidermidis* ATCC35984 (10^6^ CFU/mL in MHB) in 96-well polypropylene microtiter plates and grown for 5 days to allow for biofilm formation. Fresh media was exchanged every 24 h. After 5 days the rods were placed into fresh media and treated with APIM-peptides, water (control), or a peptide containing only the cell penetrating arginine tail of the APIM-peptide (R11). The rods were harvested 1, 6, and 24 h after the addition of peptides. Here, the rods were washed once in 0.2 mL saline solution before being transferred to 0.5 mL saline solution in sterile glass tubes. The rods were then vortexed for 20 s (2,500 rpm) followed by sonication for 6 min (40 kHz, 200W, BactoSonic^®^). The rods were again vortexed after sonication. Samples plated in duplicates on blood agar plates (10-fold dilutions in saline solution). CFU counts were calculated after 24 h incubation at 37°C. In addition, the rods were put into 3 mL fresh media after sonication and incubated 24 h (37°C, shaking) to check for visible re-growth of bacteria from the rods.

### Preparation of Bone Grafts

The preparation of the bone grafts and the bone grafts experiments are based on a bone graft model (Witso et al., 2020). Corticospongeous bone grafts were harvested from Norwegian white sheep, delivered from Nortura Malvik, Norway. Rib bones were cut to approximately 1 × 1 cm in size, height ∼0.5 cm, hollowed from end to end with a 3 mm drill through the bone marrow, creating a cylindrical hole through the bone. In addition to this, a smaller hole (2 mm wide) was drilled on the top of the bone grafts, perpendicular to the cylindrical hole in the bone marrow. Periosteum from the bone was removed and sharp edges of the bone grafts were rounded, and the bone grafts were sterilized by autoclaving. The cylindrical hole of the bone grafts was filled with bone cement with a syringe. Bone cement without gentamicin (Biomet Bone^®^ Cement R) was combined with water (control) or APIM-peptide. Gentamicin-containing bone cement (PALACOSR^®^ + G) was used as it came from the provider (adjusted with corresponding amount of water) or was combined with APIM-peptide (combination treatment). APIM-peptide was dissolved in water (160 mg/mL) before mixing it with the cement liquid component, enabling a homogenous cement containing 1.6% APIM-peptide (net peptide, 25 mg APIM-peptide/g cement, corresponding to 16 mg net peptide/g cement). The corresponding amount of water was added to the control cement without APIM-peptide. The hole on top of the bone grafts was blocked when added cement, keeping it hollow to allow the bacterial inoculum to reach the cement inside the bone graft.

### Infections of Bone Grafts *in vitro*

The bone grafts, containing cement filled with APIM-peptide, gentamicin, or a combination of these, were each added an inoculum of 50 μL TSB containing 1,000 CFU of MRSE in the hole on the top. After 24 h incubation (37°C) the bone grafts were harvested for quantification of bacterial load. The bone grafts were “opened” with a sterile scalpel to separate the cement inside from the bone. The bone and cement pieces were placed in 3 mL saline solution each (0.9% NaCl) in sterile glass tubes. Samples were sonicated for 5 min in a water bath (40 kHz, 200 W, BactoSonic^®^, Bandelin GmbH, Berlin) in addition to being vortexed 30 s (2,500 rpm) before and after sonication. Samples were plated in triplicates on blood agar plates diluted 10-fold in saline solution.

### *In vivo* Bone Infection Model

The animal study was reviewed and approved by Norwegian Food Safety Authority (Mattilsynet, application ID 11937). The animals were acclimatized for a week after arrival to the Comparative Medicine Core Facility (NTNU, St. Olav’s Hospital). The animals were housed in solitude during the experiment and had access to environmental enrichment and standard food/water *ad libitum* in a 12 h night-day cycle. The rats were weighed on the first and the last day of the experiment and only minor weight loss was observed.

The surgery of every rat was performed in a specialized cabinet with laminar airflow and in accordance with standard operating room procedures. 36 male *Rattus norvegicus* (Albino outbred Wistar) were used, 7–14 weeks old weighing from 300 to 500 g. The rats were shaved on the back before surgery. The rats were anesthetized with inhalation of isofluorane (carrier gas 66% N_2_O, 34% O_2_) delivered in an induction chamber (3–5% isoflurane) before surgery and by an inhalation mask (1.5–2.5% isoflurane) during surgery. The rats were placed on their stomach on sterile drapes covered with sterile sheets, only exposing the shaved mid part of the back. The exposed area was washed with 70% alcohol and a ∼4 cm long incision was made in the midline of the upper part of the back. One intramuscular cavity was made on each side of the back midline with a scissor. Two bone grafts were each instilled with 5 μL saline, containing 1,000 CFU of *S. epidermidis*, in the small upper hole of the bone graft, before being placed sub-fascial in the intramuscular pockets, one bone graft in each side. The incision was closed with sutures and the rats were given analgesia (Buprenorphine; Temgesic, Reckitt & Colman), 0.05 mg/kg) at the end of the surgery and postoperatively (8–12 h after the surgery).

The operation wounds of the rats grew without any signs of bacterial infection and the general health of the animals did not seem to be affected by the implanted infected bone grafts. The bone grafts were retrieved after 4 days. The animals were anesthetized, as described above, and the sutures on the back of the rats were removed and the incision made on day 1 was re-opened. The bone grafts were harvested with a sterile tweezer and a muscle biopsy (approximately 5 mm) was taken from the tissue surrounding the bone graft. The bacterial load was quantified as described in the *in vitro* bone infection model, and random bacterial colonies were analyzed with MALDI-TOF and confirmed to be the same bacterial species as inoculum. The rats were euthanized after the experiment with pentobarbital (Mebumal 10%, 0.1 mL/100 g), by intracardiac injection or intravenously injection (tail). No signs of systemic infection were found, i.e., the spleen biopsies (5–10 mm) of the rats were negative. The tissue around bone grafts was mainly unaffected by the infection and very few CFU were detected in the muscle biopsies. All biopsies were ground in 1 mL saline solution with mortar and pestle and plated on chocolate and blood agar plates.

## Results

### APIM-Peptide Increases the Efficacy of Gentamicin

Gentamicin is a widely used antibiotic against *S. epidermidis* infections. Here we selected a gentamicin susceptible *S. epidermidis* strain and a gentamicin resistant MRSE strain for MIC testing of APIM-peptide, gentamicin and the combination of these. In addition, the ATCC 35984 *S. epidermidis*, which is producing biofilm, was tested for gentamicin and APIM-peptide sensitivity. Table 1 shows that MIC for APIM-peptide was 4 and 5 μM for the gentamicin susceptible *S. epidermidis* and the MRSE strain, respectively. The MIC for gentamicin was 0.72 μM for the susceptible strain and 309 μM for the MRSE strain. In combination with APIM-peptide; however, the MICs for gentamicin was reduced three and eightfold, respectively. The ATCC 35984 strain also showed reduced MIC for gentamicin when combined with 0.5x MIC of APIM-peptide. This indicates that the combination of APIM-peptide and gentamicin has an additive effect.

**TABLE 1:** *S. epidermidis* sensitivity toward APIM-peptides and gentamicin.

Strain	Agent	MIC (μM)	MIC combination (μM)
*S. epidermidis*	APIM-peptide	4	2
	Gentamicin	0.72	0.24
MRSE	APIM-peptide	5	3
	Gentamicin	309	39
ATCC 35984	APIM-peptide Gentamicin	3 248–496	1.5 62–124

**MIC values for three S. epidermidis strains: one gentamicin sensitive and one gentamicin resistant (MRSE) clinical isolate, and the reference strain ATCC 35984. MIC tests were performed with APIM-peptides and gentamicin alone and in combination. Representative data from minimum 3 experiments are given. MRSE, methicillin resistant S. epidermidis; MIC, Minimal inhibitory concentration.**

### APIM-Peptide Rapidly Kills MRSE

**FIGURE 1:**
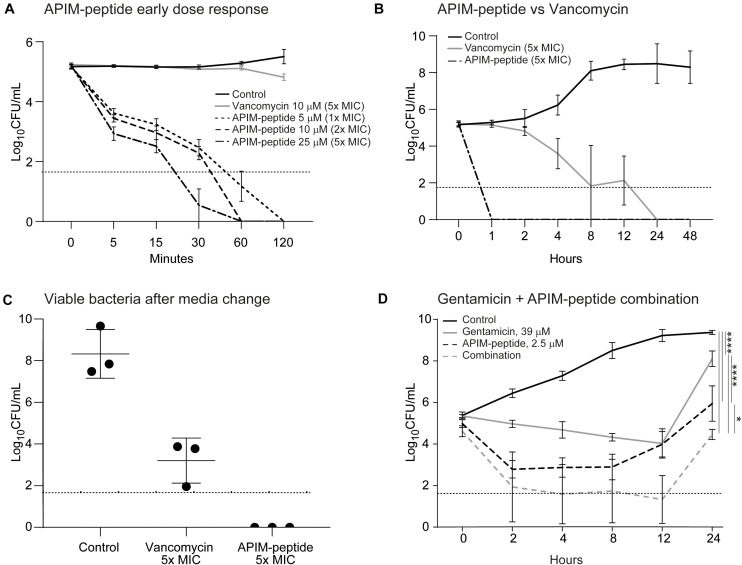
Time killing assays and viable bacteria after media exchange of MRSE cultures. Addition of APIM-peptides, vancomycin or gentamicin conducted at time point 0. The control was added the corresponding amount of water. The dotted horizontal line shows the detection limit of CFU/mL, but when no colonies were found, 0 is used for calculation of the average. **(A)** Time-kill assay with early dose-response with 5, 10, and 15 μM (1x, 2x, and 5x MIC) of APIM-peptide compared to 5x MIC of vancomycin (10 μM, MIC = 2 μM). **(B)** Time-kill assay with a later response (up to 48 h) of cultures added 5x MIC of APIM-peptide and vancomycin. **(C)** Viable bacteria after media exchange, 48 h after addition of 5x MIC APIM-peptide and 5x MIC vancomycin. **(D)** Time-kill assay with cultures grown 24 h after addition of APIM-peptide (1/2x MIC, 2.5 μM) and/or 39 mM gentamicin (MIC = 309 μM). Two-way ANNOVA, multiple comparison, ****p < 0.0001, *p < 0.05.

To assess the bactericidal activity of APIM-peptide compared to other antibacterial drugs, a time-kill experiment was conducted. CFU counts from the MRSE culture were determined from 5 min and up to 48 h after the addition of APIM-peptides, vancomycin, or gentamicin. The time-kill experiment showed that the addition of all three APIM-peptide doses caused a significant decrease in survival already after 5 min (Figure 1A); 1x MIC (5 μM) of APIM-peptide killed 97% of the bacteria after 5 min and has complete bactericidal effect (defined as ≥ 3log_10_ decrease of original inoculum) (NCCLS., 1999) after 60 min. APIM-peptides at 2x and 5x MIC have even faster killing efficiencies, i.e., bactericidal effect was seen after 30 min with 5x MIC. In comparison, a bactericidal effect with 5x MIC vancomycin (10 μM) addition was first detected 8 h after addition (Figure 1B). Vancomycin is known to be bacteriostatic at lower doses on staphylococci (Stevens, 2006), and this was also seen in our MIC assay using 1x MIC (Supplementary Figure S1). Both 5x MIC of APIM-peptide and vancomycin inhibit bacterial growth up to 48 h without any re-growth; however, when removing the antibiotics by transferring the bacteria into fresh media after 48 h, CFU determinations revealed bacteria (∼4.5 × 10^3^ CFU/mL) in the vancomycin culture but not in the APIM-peptide culture (Figure 1C).

To further investigate the additive effect of the combination, APIM-peptide (2.5 μM, 0.5x MIC) was combined with gentamicin (sub-MIC, 39 μM) in a time-kill experiment with the MRSE strain. The combination gave a 1.5-log_10_ decrease in bacterial growth compared to single-agent addition with APIM-peptide at 24 h (Figure 1D).

### APIM-Peptide Inhibits *S. epidermidis* Biofilm Formation and Eradicates Existing Biofilm

**FIGURE 2:**
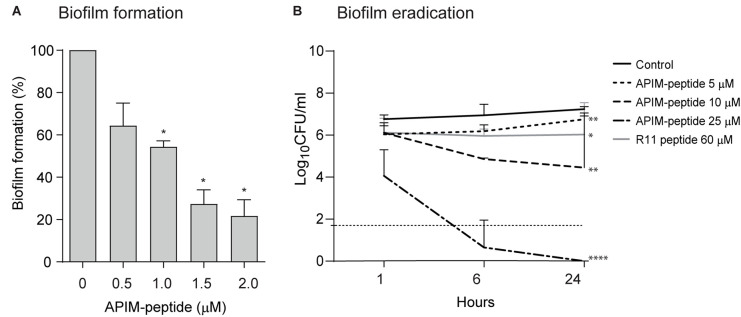
Effect of APIM-peptides on development and eradication of *S. epidermidis* biofilm. **(A)** Christensen’s biofilm microtiter plate test using *S. epidermidis* ATCC 35984. Sub-MIC doses of APIM-peptide (0.5–2 μM) and water (control) added at the same time as the bacteria. Biofilm formation assessed after 24 h. Average biofilm formation from three independent experiments, minimum 3 wells per sample, are shown. Two-tailed, paired Student’s *t*-test *p < 0.05. **(B)** Biofilm killing experiment. Biofilm was established on steel rods by growing ATCC 35984 for 5 days before addition of 5 (MIC), 10, and 25 μM of APIM-peptide, and 60 μM of the cell penetrating peptide in the APIM-peptide, R11. CFU determination of the steel rods 1, 6, and 24 h after peptide addition, plotted as average from three independent experiments, are shown. The dotted horizontal line shows the detection limit of CFU/mL, but when no colonies were found, 0 is used for calculation of the average. Two-tailed, unpaired Student’s *t*-test at 24-h timepoint, *p < 0.05, **p < 0.01, ****p < 0.0001.

Because biofilm formation is an important parameter in chronic *S. epidermidis* infections, the effects of APIM-peptide on the formation and eradication of biofilm were tested. The MRSE strain used in the previous *in vitro* studies described above is a relatively poor biofilm former (Supplementary Figure S2); thus, for these experiments the biofilm-forming reference strain ATCC 35984 was applied. Christensen’s microtiter plate biofilm assay showed that APIM-peptide reduces the formation of biofilm up to ∼80% at 2 μM, which equals ∼0.5x MIC (Figure 2A). To test if APIM-peptide has an effect on already existing biofilm, we grew ATCC 35984 biofilm on C-wire steel rods, added APIM-peptide and quantified CFU after sonication of the rods. Here, 25 μM (∼8x MIC) of APIM-peptide completely eradicated the biofilm (Figure 2B) and no re-growth after transferring the rods to fresh media was detected (data not shown). Lower doses of the peptide also significantly reduced the biofilm, i.e., the doses of 5 and 10 μM APIM-peptide resulted in ∼0.5 and ∼3 log decrease in CFU/mL, respectively (Figure 2B). To exclude that this anti-biofilm effect was due to the cell penetrating part of the APIM-peptide, we included the 11-arginine cell penetrating peptide, R11, as a control. However, 60 μM of the R11 peptide gave only ∼1 log decrease in CFU/mL, showing that, similarly to the antibacterial and anti-mutagenic effects (Nedal et al., 2020), the full-length peptide containing APIM is needed for maximal anti-biofilm effect. Preliminary data also shows that coating steel rods with APIM-peptide dissolved in polyethylene glycol (PEG) inhibited biofilm formation (data not shown); thus, APIM-peptides could potentially be exploited for further use in coating of surgical implants.

### APIM-Peptide Is Released From Bone Cement and Has a Strong Antibacterial Effect in an *in vivo* Bone Infection Model in Combination With Gentamicin

To test APIM-peptide in a clinically relevant *in vivo* model related to PJI, i.e., an infected prosthesis, a variant of a bone graft model in rats was used (Material and Methods; Witso et al., 2020). Hollow bone grafts were filled with cement containing APIM-peptide, gentamicin, or a combination of these, thereby functioning as a local vehicle for delivery of the antibacterial agents. The bone grafts were infected with MRSE to mimic the event of bacterial infection colonizing the grafts at the time of surgery.

In initial *in vitro* experiments, APIM-peptide was shown to be rapidly released/eluted from the cement (see Supplementary Figure S3A). Further, *in vitro* studies using bone grafts infected with MRSE also showed that the APIM-peptide alone and in combination with gentamicin abolished bacterial growth. The cement was separated from the bone to quantify the bacterial load on each part of the bone grafts. All the treatments reduced bacterial growth in the bone compared to the control *in vitro*; however, the cement containing only gentamicin had some bacterial growth (Supplementary Figures S3B,C).

**FIGURE 3:**
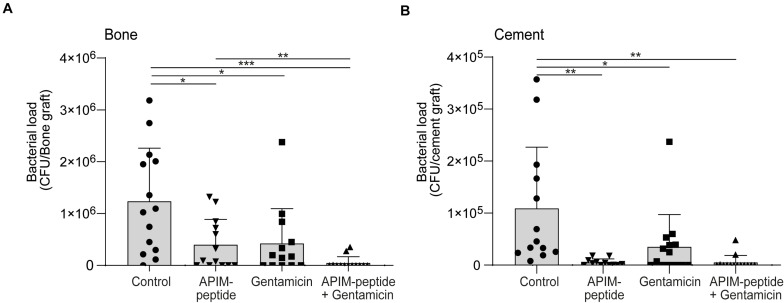
Antibacterial effect of APIM-peptide and gentamicin in an *in vivo* bone graft model. MRSE growth in infected bone grafts filled with bone cement containing APIM-peptide, gentamicin, and a combination of gentamicin and APIM-peptide or water (control). Bone grafts were infected (10^3^ CFU inoculum), inserted intramuscularly in rats (2 per animal), and retrieved after 4 days. The bone graft and the cement pieces inside were removed and bacterial load was quantified separately in bone and cement. Student two tailed-paired *t*-test, only statistically significant differences are depicted in the figure, *p < 0.05, **p < 0.01, ***p < 0.001. **(A)** CFU count of the bone grafts. **(B)** CFU count of the bone cement pieces.

Next, infected bone grafts were next implanted into the back of rats and harvested after 4 days. The quantification showed that the bacterial load was 96% reduced in bone grafts containing cement with the APIM-peptide-gentamicin combination, compared to control (Figure 3A). In bone grafts treated with APIM-peptide, the bacterial load was reduced by 68% compared to control, whereas gentamicin alone reduced the bacterial load by 66%. Calculating the fraction of animals with detectable bacterial growth vs. no growth in the bone grafts, showed that 77% of the bone grafts containing cement with the combination had no detectable growth, vs. 38% in the groups containing either APIM-peptide or gentamicin.

The bacterial load on the bone cement (Figure 3B) correlates with the bacterial load found in the bone (Figure 3A), except for the detection of an even lower bacterial load on the cement containing only APIM-peptide. All the additions to the cement significantly reduced bacterial load compared to control (Figure 3B). There is a tendency that the cement containing APIM-peptide (either alone or in combination) have lower bacterial load than cement with only gentamicin; however, this is significant (*p* < 0.05) only if the outlier in the gentamicin group is removed. Removing the similar outlier in the gentamicin group in Figure 3A, also yields a significant difference between gentamicin and the combination.

## Discussion

The growing problem of antibacterial resistance highlights the need for new antibiotics with a novel mode of action. In this paper, the recently discovered antibacterial effect of APIM-peptides (Nedal et al., 2020) and their potential use in a clinical setting with MRSE infections are explored. The results show that APIM-peptide kill *S. epidermidis* rapidly in *in vitro* planktonic cultures as well as in biofilms. In addition, APIM-peptide combined with gentamicin in bone cement nearly eliminate the bacterial load in infected bone grafts *in vivo*.

The combination of APIM-peptide and gentamicin showed an additive effect on both gentamicin sensitive and resistant *S. epidermidis* strains. This effect could be explained by different modes of action of APIM-peptide and gentamicin, i.e., targeting DNA and protein synthesis, respectively (Block and Blanchard, 2019; Nedal et al., 2020). The eight-fold reduction in MIC for gentamicin when combined with APIM-peptide in the gentamicin resistant strain suggests that the APIM-peptide could re-sensitize bacteria toward antibiotics. The ATCC 35984 strain showed variable MICs of gentamicin (Table 1, size range from 4 experiments are given). This could be due to its biofilm forming properties. However, the gentamicin MIC was reduced 2–8 times in all experiments when combined with 0.5x MIC APIM-peptide. Re-sensitizing of resistant bacteria toward erythromycin and azithromycin when combined with APIM-peptides have also been observed for MRSA strains (unpublished results). In addition, APIM-peptides are shown to lower the mutation frequency in *E. coli* and to inhibit resistance development toward gentamicin in *S. epidermidis* (Nedal et al., 2020). Combinations with APIM-peptides should be further explored as it can potentially be used to both reduce mutagenesis, and thereby prevent development of resistance, and increase the effects of other antibiotics.

Bactericidal effect of the APIM-peptide in planktonic MRSE cultures was already detected after 30 min incubation (Figure 1A), while it took 8 h before vancomycin completely killed the planktonic culture (Figure 1B). An engineered cationic antimicrobial peptide called WLBU2, now in phase 1 trials for treatment of PJIs (Deslouches et al., 2020), has shown comparable effect as APIM-peptides on staphylococcus with regard to rapid bactericidal effects; *S. aureus* where killed at 2x MIC already after 10 min (Mandell et al., 2017). We detected re-growth in less than 50% of the time-killing experiments using 1x MIC of APIM-peptide (Supplementary Figure S1); however, this is likely due to slightly different assay conditions than in the MIC-assay. The rapid bactericidal effect of the APIM-peptides, and the ability to completely eradicate all CFUs in culture, together with the additive effect observed in combination with gentamicin, could be important traits for clinical application and for inhibiting the development of antibacterial resistance.

*S. epidermidis* utilize and grow biofilm to manifest and colonize on surgical grafts; thus, it is important to inhibit the formation of biofilms as early as possible (Howlin et al., 2015). The ability of APIM-peptide to inhibit biofilm formation in sub-MIC concentrations (Figure 2A) could indicate that the APIM-peptide inhibits the cells’ ability to adhere. This is also supported by the *in vivo* bone graft model where the cement containing APIM-peptide had significantly fewer bacteria than the cement containing no antibiotic or gentamicin only (Figure 3B). It has been shown that *S. epidermidis* can persist on gentamicin-loaded cement despite being susceptible to gentamicin, and that susceptible strains can thereby develop resistance against gentamicin (Anagnostakos et al., 2008). If APIM-peptides inhibit the bacteria adhering to surfaces or if this is merely due to the bactericidal effect of APIM-peptides, is yet to be determined. The effect of APIM-peptide on already existing biofilm could also be explained by the bactericidal effect of the APIM-peptide seen in planktonic cultures, but it can also indicate that the APIM-peptide has effects on slow-growing/dormant cells. The latter is supported by no regrowth of dormant cells after APIM-peptide addition followed by media exchange (Figure 1C). The complete elimination (and no re-growth) of biofilm by the APIM-peptide (Figure 2B) is an important characteristic as many traditional antibiotics have been unable to completely remove the biofilm on surgical implants (Otto, 2018). The WLBU2 peptide has also shown anti-biofilm effect on *S. aureus*, where 10x MIC eliminated *in vitro* biofilm (Mandell et al., 2017). This is comparable with APIM-peptide where ∼8x MIC eliminated the *S. epidermidis* biofilm.

The *in vivo* study showed an increased antibacterial effect when combining APIM-peptide and gentamicin in surgical cement. A rapid elution of APIM-peptide from the cement would lead to high local concentrations of the peptide; however, no sign of toxicity was observed in the surrounding tissue in the animals.

It could be argued that the incubation time (4 days) is too short for miming a chronic infection, but this harvest time was selected from previous studies with a bone graft model without cement, where the same bacterial inoculum was found to grow quickly up to a certain point and then remain stable for up to 42 days, mimicking a chronic infection (Witso et al., 2020). From pilot experiments using our model, we found that 4 days yielded a sufficient number of bacteria for detecting the effect of the treatments (data not shown). Still, our results are from a simplified animal model using only one MRSE strain; thus, these results cannot directly be transferred to clinical PJI situations.

Another aspect to consider if the APIM-peptides are to be used in the clinic is that addition of antibiotics to bone cement can alter the mechanical properties of the cement. The cement used in these experiments, Palacos R + G, contains 1.25% gentamicin (w/w), and we added additional 2.5% total mass APIM-peptides-salt (w/w, net-peptide; 1.6%). Thus, the total amount of both gentamicin and APIM-peptides adds up to 2.86–3.75%. This is within the recommended amount of additives in Palacos^®^ R cement (≤6.5%) in order to retain the mechanical and elution properties of the cement (He et al., 2002). The APIM-peptide as a powder is very stable (>3 years at 4°C) enabling the possibility to add APIM-peptide directly to the cement powder. The type of cement and antibiotic constituent can have different effects on the mechanical strength of the cement (Galvez-Lopez et al., 2014) and both the stability of the APIM-peptide in cement and its effects on mechanical strength of the cement remains to be tested. Still, with all these limitations taken into account, the results from the *in vivo* bone graft model showed that the APIM-peptides inhibited bacterial growth in bone grafts and increased the effect of gentamicin; thus, APIM-peptides could be a promising antibacterial drug candidate for PJIs preventions as additives to bone cement.

## Conclusion

Our results showed a good antibacterial effect of APIM-peptide on *S. epidermidis.* APIM-peptide showed a bactericidal effect *in vitro* and was rapidly eluted from bone cement. A greatly reduced bacterial load was seen *in vivo* in a bone infection model when combining APIM-peptide and gentamicin in bone cement. In addition, APIM-peptide inhibits the development of biofilm and can eliminate existing biofilms. These results supports that APIM-peptides could be a promising new drug candidate to use in antibiotic-loaded bone cement for treatment of PJIs.

## Data Availability Statement

The raw data supporting the conclusions of this article will be made available by the authors, without undue reservation.

## Ethics Statement

The animal study was reviewed and approved by the Norwegian Food Safety Authority (Mattilsynet).

## Author Contributions

SR, ES, EW, and MO planned and initiated the study. SR, ES, EW, AN, KL, and KB performed the laboratory experiments and interpreted the results. SR and MO wrote the manuscript. All authors contributed to the article and approved the submitted version.

## Conflict of Interest

The authors declare that the research was conducted in the absence of any commercial or financial relationships that could be construed as a potential conflict of interest.

## Footnotes

1 Hancock Laboratory Methods, http://cmdr.ubc.ca/bobh/method/modified-mic-method-for-cationic-antimicrobial-peptides/

## Abbreviations

APIM: AlkB homolog 2 PCNA-interacting motif
MIC: Minimal inhibitory concentration
MRSA: Methicillin resistant *Staphylococcus aureus*
MRSE: Methicillin resistant *Staphylococcus epidermidis*
PCNA: Proliferating cell nuclear antigen
PJIs: Prosthetic joint infections.
