# Supplementary material for: Novel Peptides Targeting the β-Clamp Rapidly Kill Planktonic and Biofilm Staphylococcus epidermidis Both in vitro and in vivo
Source: Front Microbiol. 2021 Mar 17;12:631557. doi: 10.3389/fmicb.2021.631557 (PMC8009970; doi:10.3389/fmicb.2021.631557)
Supplement: Supplementary file 1 [file Presentation_1.pdf]

Supplementary Material

Novel peptides targeting the β-clamp rapidly kill planktonic and biofilm Staphylococcus epidermidis both *in vitro* and *in vivo*

1 Supplementary Figures

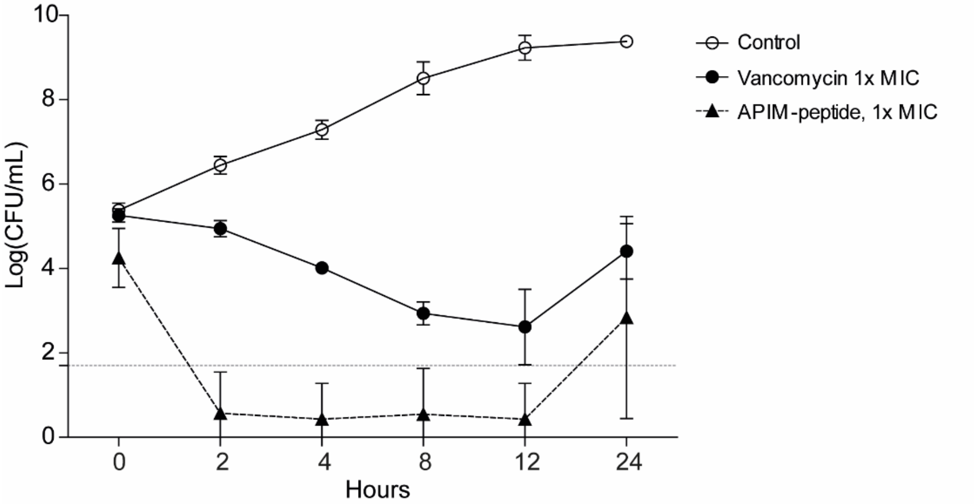

Supplementary Figure S1. Time-kill assay with MRSE cultures added 1x MIC APIM-peptide or vancomycin. Cultures were added vancomycin or APIM-peptide (3 mg/L and 5 µM respectively) at 0h and plated to quantify bacterial density (CFU/ml) at timepoints up to 24 hours. Data included are from three independent experiments. The dotted horizontal line shows the detection limit of CFU/mL, but when no colonies were found, 0 is used for calculation of the average.

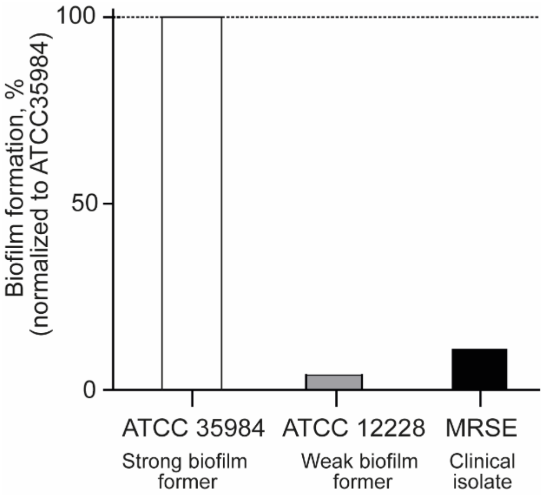

Supplementary Figure S2. Relative biofilm formation of different *S. epidermidis* strains. *S. epidermidis* strain used in bone graft model (MRSE) and a poor biofilm former ATCC12228, was compared to the biofilm forming strain, ATCC 35984, in a biofilm formation assay, where biofilm was measured 24 hours after seeding of cultures. n=1.

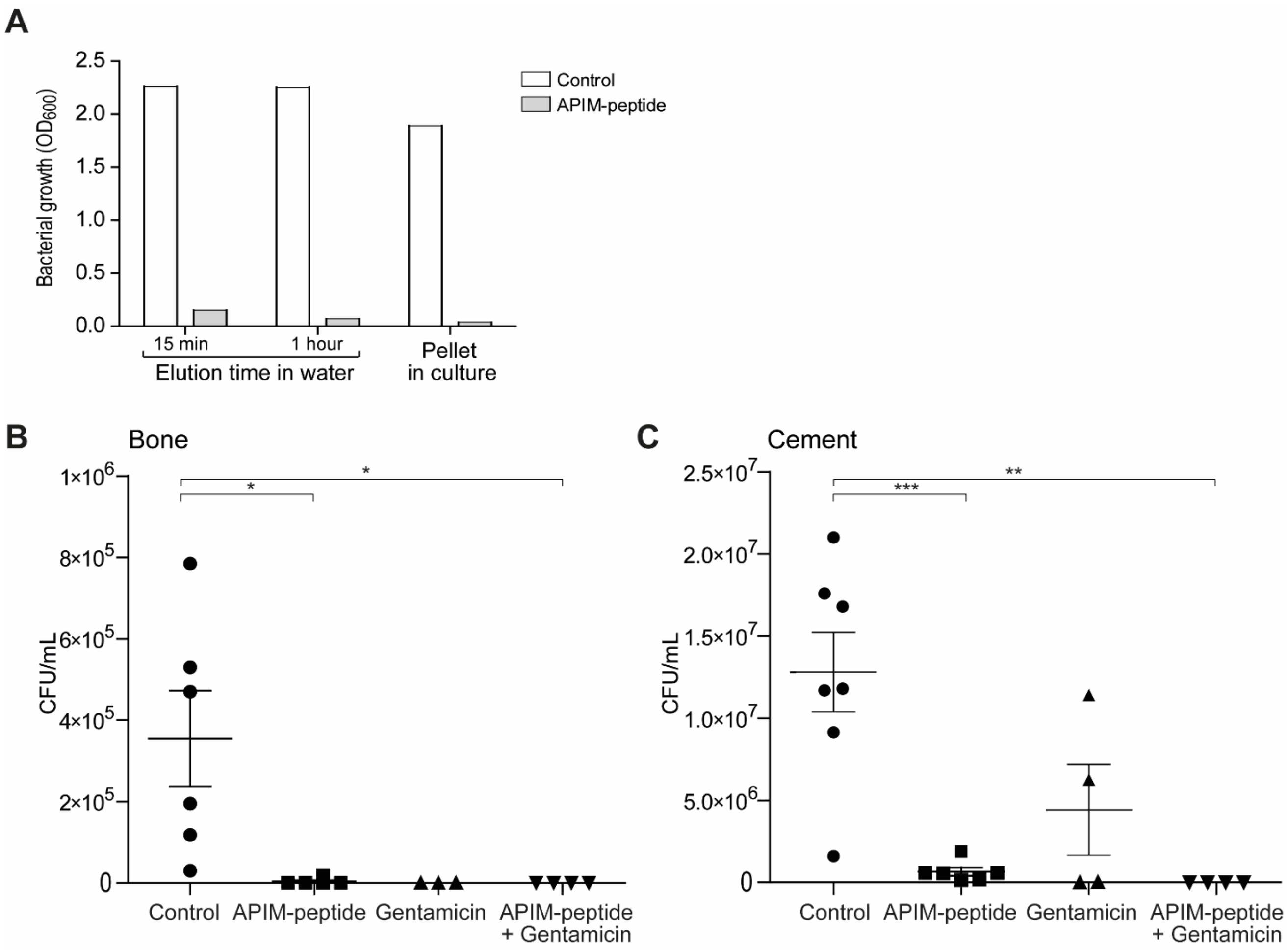

Supplementary Figure S3. The effect of eluted APIM-peptide from surgical cement on bacterial growth in culture and in a bone graft model in vitro. (**A**) To examine if APIM-peptide is eluted from bone cement, cement pellets without additions (control) or APIM-peptide were incubated in 100 µL water for 15 minutes or 1 hour, before this water was added to MRSE cultures containing. MRSE cultures were also added cement pellet directly (pellet in culture). Cultures were grown for 24 hours before culture density was measured (absorbance, OD600). (**B-C**) Quantification of MRSE bacterial load in infected bone grafts filled with bone cement containing APIM-peptide, gentamicin or a combination. Control bone grafts contain bone cement without additions. 24 hours after infection the bone grafts (**B**) and the cement inside (**C**) were separated and bacterial load was quantified. Student two tailed unpaired t-test, only statistically significant differences are depicted in the figure, *=$p<0.05$ **=$p<0.01$, ***=$p<0.001$. The dotted horizontal line shows the detection limit of CFU/mL, but when no colonies were found, 0 is used for calculation of the average.
